# Preoperative Surgical-Field Antisepsis with a pH-Optimized Sodium Hypochlorite Wound Cleanser in Microsurgical Diabetic Foot Reconstruction: A Single-Center Feasibility Study

**DOI:** 10.3390/jcm15166272

**Published:** 2026-08-13

**Authors:** Junho Lee, Sarah Kim, Jung Ho Lee, Daiwon Jun

**Affiliations:** 1 Department of Plastic and Reconstructive Surgery, Uijeongbu St. Mary’s Hospital, College of Medicine, The Catholic University of Korea, Seoul 06591, Republic of Korea; drizawa88@gmail.com; 2 Department of Plastic and Reconstructive Surgery, Bucheon St. Mary’s Hospital, College of Medicine, The Catholic University of Korea, Seoul 06591, Republic of Korea; sarahkim9511@gmail.com (S.K.); tfm0822@gmail.com (J.H.L.)

**Keywords:** diabetic foot ulcer, microsurgical reconstruction, sodium hypochlorite, biofilm, surgical site infection, feasibility study

## Abstract

**Background/Objectives:** Postoperative infection is the leading cause of limb loss after microsurgical reconstruction of diabetic foot ulcers (DFUs), and povidone-iodine alone neither disrupts wound-bed biofilm nor penetrates hyperkeratotic debris. We assessed the feasibility of whole-field preoperative antisepsis with a pH-optimized sodium hypochlorite (NaOCl) cleanser applied before povidone-iodine draping. **Methods:** Consecutive patients undergoing microsurgical DFU reconstruction (January 2023–May 2024) were grouped by date of protocol introduction into pre-protocol (*n* = 9) and post-protocol (n = 6) cohorts. Primary outcome: technical feasibility, assessed by protocol completion, operative time, flap survival, limb salvage, and flap revision. Secondary, descriptive: postoperative infection, early (≤30 days) or late (>30 days). **Results:** The protocol was delivered as specified in all six post-protocol patients, without procedural modification or attributable adverse event. Operative time did not differ (388 vs. 430 min; *p* = 0.596); flap survival and limb salvage were 100% in both groups, with one flap revision per group. Baseline characteristics were comparable. Early infection was similar (16.7% vs. 11.1%; *p* = 1.000); late infection was absent post-protocol versus 33.3% pre-protocol (OR 0.14, 95% CI 0.01–3.4); and total infection was 16.7% versus 44.4% (OR 0.33, 95% CI 0.04–3.0). Follow-up was asymmetric (4.5 vs. 24.5 months); a 6-month landmark analysis showed 0 of 3 versus 3 of 6 (*p* = 0.464). **Conclusions:** Whole-field preoperative NaOCl antisepsis was technically feasible, with no adverse effect on flap survival or limb salvage. No statistically significant differences in the parameters were detected, though the sample is too small to establish comparability. An adequately powered trial is required.

## 1. Introduction

Diabetic foot ulcer (DFU) is among the most serious complications of diabetes mellitus, affecting approximately 19–34% of patients with diabetes over their lifetime and accounting for the majority of non-traumatic lower-limb amputations worldwide [1,2]. The prognosis following major lower extremity amputation is devastating, with five-year mortality exceeding 50%, a burden comparable to that of many common malignancies [3]. Microsurgical free tissue transfer has emerged as a pivotal limb-salvage strategy for complex DFUs with exposed bone, tendon, or joint, achieving flap survival rates of 92% and limb salvage rates of 83–85% in dedicated centers [4,5]. Nevertheless, reconstruction in this population carries substantially higher complication rates than in non-diabetic patients, with major complications requiring operative reintervention reported in up to 19% of patients [6].

Postoperative infection occupies a unique role among the complications of microsurgical DFU reconstruction. In a representative series of 29 patients undergoing free tissue transfer for recalcitrant DFUs, five of seven eventual major amputations were attributable to recalcitrant infection rather than vascular compromise or flap failure per se [4]. Late-onset infections, those occurring beyond 30 days, are particularly insidious: polymicrobial, biofilm-mediated, and often refractory to systemic antibiotic therapy alone. Diabetic patients are intrinsically susceptible to wound infection through multiple mechanisms, including impaired neutrophil chemotaxis and oxidative burst activity, glycation of structural proteins, and peripheral neuropathy that delays symptom recognition [7].

Clinical evidence for NaOCl- and HOCl-based cleansers in the diabetic foot is growing. The first randomized trial of diluted NaOCl versus standard saline care in infected DFUs demonstrated ulcer closure in 35.6% versus 4.4% of control patients, lower amputation rates (2.2% versus 28.9%), and substantially reduced bacterial burden (all *p* < 0.001) [8], and a retrospective series reported limb salvage in 95.8% of patients with severe diabetic foot infection treated with cyclical NaOCl instillation, without impairment of wound healing [9].

Two mature bodies of evidence approach the question addressed here from different directions but do not meet. The first establishes HOCl/NaOCl as an effective wound-bed treatment in the diabetic foot, validated against clinical outcomes, as reviewed above. The second establishes the value of antiseptic preparation of the surgical field, with network meta-analytic evidence that intraoperative antiseptic irrigation reduces surgical-site infection relative to saline or no irrigation, while saline itself confers no benefit [10,11,12]. What neither addresses is the combination: preoperative antisepsis of the open, biofilm-laden wound bed together with the surrounding at-risk skin, evaluated against a surgical infection endpoint in diabetic foot free-flap reconstruction. The closest analog is a recent series applying HOCl wound-bed preparation before flap closure of chronic stage IV pressure injuries [13]; no comparable evaluation exists for whole-field preoperative antisepsis in the diabetic foot.

This retrospective comparative feasibility study was conducted to determine whether preoperative antisepsis of the entire surgical field (the open wound bed together with the surrounding foot and lower leg) with a pH-optimized sodium hypochlorite (NaOCl)-based wound cleanser applied before conventional povidone-iodine draping is feasible in microsurgical DFU reconstruction, and to characterize postoperative infection outcomes under this protocol.

## 2. Materials and Methods

### 2.1. Study Design and Ethical Approval

This was a single-institution, retrospective, comparative feasibility study. The study was approved by the Institutional Review Board (HC26RISI0076). The requirement for individual informed consent was waived under the institutional policy for retrospective chart reviews. All procedures were conducted according to the principles of the Declaration of Helsinki.

### 2.2. Study Period and Sequential Design

Patients were identified from the operative database from January 2023 to May 2024. Preoperative surgical field antisepsis with the pH-optimized NaOCl-based cleanser was introduced into the operative protocol of the senior author (D.J.) in November 2023. Patients operated before this date (January to September 2023) were assigned to the pre-protocol group; those operated from November 2023 onwards constituted the post-protocol group. This sequential allocation reflects the chronological adoption of the institutional protocol rather than prospective randomization.

### 2.3. Patient Selection

Patients were eligible if they (1) had type 2 diabetes mellitus, (2) presented with an active DFU with exposure of vital structures such as bone, tendon, joint or neurovascular structures requiring soft tissue reconstruction, and (3) underwent microsurgical free-flap reconstruction during the study period. Patients were excluded if the indication for reconstruction was post-ulcer wound contracture or scar deformity without an active ulcer (n = 1 excluded). No restrictions were applied based on comorbidity burden, renal replacement status, or peripheral arterial disease.

### 2.4. Preoperative Surgical Field Antisepsis Protocol

All post-protocol patients received preoperative antisepsis of the entire surgical field with a pH-optimized sodium hypochlorite (NaOCl)-based wound cleanser (Meditouch^®^ Wound Solution, Ildong Pharmaceutical, Seoul, Republic of Korea). The solution was applied by wipe/swab technique using sterile gauze saturated with the solution, immediately prior to povidone-iodine skin preparation and draping (Supplementary Video S1). Approximately 100 mL was used in a single application session. Application comprised (i) cleansing of the open wound bed and (ii) wipe/swab passes over the surrounding foot and lower leg. Contact time was not prospectively recorded and could not be reconstructed from retrospective review. No sodium hypochlorite solution was instilled intraoperatively at any stage. Pre-protocol patients received povidone-iodine preparation and draping without preoperative cleansing. Following surgical debridement, both cohorts received routine saline irrigation of the wound.

### 2.5. Operative Technique

All reconstructions were performed by the senior author (D.J.). Following surgical debridement with a dedicated instrument set, a separate sterile instrument set was used for the reconstructive phase to prevent re-inoculation of the debrided wound [14]. Flaps included ALT, MSAP, SIEA, AMT perforator flaps, and VRAM. Microvascular anastomoses were performed under an operating microscope (OPMI Pentero Infrared 800; Carl Zeiss, Oberkochen, Germany). All patients received perioperative antibiotic therapy directed by preoperative wound culture in consultation with the infectious disease service.

### 2.6. Data Collection

Data were retrospectively collected from the institutional electronic medical records. Variables included demographics (age, sex, BMI), metabolic status (HbA1c within three months prior to surgery), duration of diabetes, comorbidities, wound severity (Wagner grade), presence of osteomyelitis, perfusion status (ankle-brachial index and angiographic findings), revascularization prior to reconstruction, perioperative antibiotic regimen, operative parameters, and outcomes (postoperative infection, flap survival, flap revision, limb salvage, new ulcer formation, follow-up duration, and vital status). Wound cultures were obtained from tissue and/or bone. Sex was not an eligibility criterion.

### 2.7. Outcome Definitions

The primary outcome was technical feasibility. Feasibility was assessed by protocol completion, operative time, flap survival, limb salvage (preservation without major amputation at or above the ankle at last follow-up), and flap revision. Because feasibility concerns delivery of the intervention, it is assessed in the post-protocol group only; the pre-protocol group provides comparatives for the descriptive outcomes. Secondary outcomes were postoperative infection rate and new ulcer formation. Postoperative infection was defined as clinical signs (erythema, wound dehiscence, purulent discharge, or fever) accompanied by either a positive wound culture or a clinical decision to institute antibiotic therapy, and classified as early (≤30 days) or late (>30 days) by time from reconstruction. Late infections are reported as new infectious episodes at the reconstructed site. Postoperative cultures were obtained only when clinical infection was suspected. Isolates were characterized to species level with antimicrobial susceptibility testing as part of routine clinical microbiology; molecular strain typing was not available at the study institution. Outcome ascertainment was performed by the operating surgeon and was not blinded to group allocation. Follow-up duration was recorded to the nearest month from the date of reconstruction.

### 2.8. Statistical Analysis

Nonparametric statistics were applied throughout. Continuous and ordinal variables are presented as median (range), and categorical variables as frequency and percentage. Between-group comparisons used the Mann–Whitney *U* test for continuous or ordinal variables and Fisher’s exact test for categorical variables. Odds ratios were calculated for binary outcomes. A two-sided threshold of *p* < 0.05 was applied. Analyses used IBM SPSS Version 23.0 (IBM Corp., Armonk, NY, USA).

## 3. Results

### 3.1. Patient Characteristics

**Figure 1:**
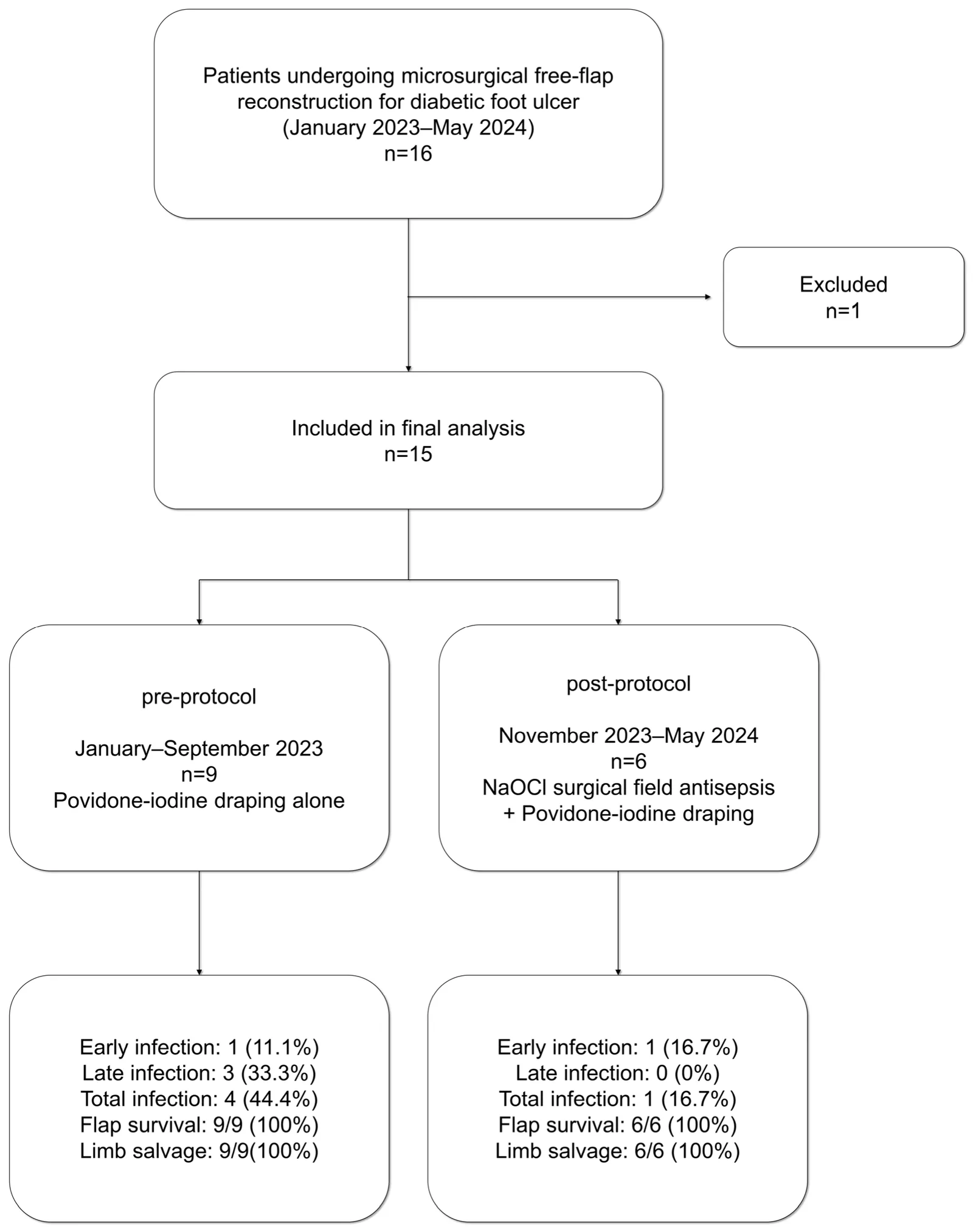
Study flow diagram. Patients undergoing microsurgical free-flap reconstruction for diabetic foot ulcer were allocated to the pre-protocol or post-protocol group by the November 2023 introduction of the preoperative surgical-field antisepsis protocol. NaOCl, Sodium hypochlorite.

Fifteen patients were included in the final analysis (post-protocol group n = 6; pre-protocol group n = 9) following exclusion of one patient whose indication for reconstruction was post-ulcer wound contracture rather than an active DFU (Figure 1). All patients were male with type 2 diabetes. Patient demographics are summarized in Table 1. The post-protocol group was numerically older than pre-protocol patients (median 70 years, range 61–80, versus median 59 years, range 34–81; *p* = 0.181). Glycemic control was closely matched (HbA1c median 8.3%, range 6.6–11.3, versus median 8.2%, range 6.1–13.7; *p* = 0.860). BMI (median 23.1 versus 22.2 kg/m^2^; *p* = 0.529) was comparable, as was wound severity by Wagner grade (*p* = 0.742). Prevalence of hypertension, smoking, peripheral arterial disease, end-stage renal disease, and coronary artery disease did not differ significantly between groups (all *p* ≥ 0.608).

### 3.2. Operative Parameters

Operative parameters are detailed in Table 2. The median flap size was 60 cm^2^ (range 28–120) in the post-protocol group and 96 cm^2^ (range 32–240) in the pre-protocol group (*p* = 0.262). Median operative time was 388 min (range 318–680) in the post-protocol group versus 430 min (range 328–615) in the pre-protocol group (*p* = 0.596). Median follow-up was 4.5 months in the post-protocol group (range 2–24) and 24.5 months in the pre-protocol group (range 3–37; one patient lost to follow-up), reflecting the retrospective sequential design.

### 3.3. Postoperative Infection

Early postoperative infection (≤30 days) occurred in one of six post-protocol patients (16.7%) and one of nine pre-protocol patients (11.1%; OR 1.55, 95% CI 0.13–18.9; *p* = 1.000); both were flap infections presenting at 9 and 11 days respectively. Late postoperative infection (>30 days) occurred in none of the post-protocol patients (0%) compared with three of nine pre-protocol patients (33.3%; OR 0.14, 95% CI 0.01–3.4; *p* = 0.229). All three late episodes were deep tissue infections arising at the reconstructed site, and occurred at 5.0, 5.2, and 8.3 months after reconstruction. The total postoperative infection rate was 16.7% in the post-protocol group versus 44.4% in the pre-protocol group (OR 0.33, 95% CI 0.04–3.0; *p* = 0.580). Because follow-up differed substantially between groups (post-protocol median 4.5 months, range 2–24; pre-protocol median 24.5 months, range 3–37; *p* = 0.060), a landmark analysis was performed, restricted to patients observed for at least six months. Six months was chosen as the shortest window that contains observed late-infection events. Within this window, infection occurred in none of the three post-protocol patients and three of the six pre-protocol patients (*p* = 0.464). Incidence expressed per person-time of observation was near-identical between groups (post-protocol 2.27 versus pre-protocol 2.33 events per 100 person-months). None of these comparisons reached statistical significance.

### 3.4. Microbiological Findings

Preoperative wound cultures were positive in five of six post-protocol patients (83.3%) and seven of nine pre-protocol patients (77.8%), indicating a comparable baseline microbiological burden; one post-protocol patient showed no growth, and two pre-protocol patients had no preoperative culture on record. Growth was polymicrobial in five of the twelve culture-positive patients. Preoperative isolates in the post-protocol group included *Stenotrophomonas maltophilia* in two patients and *Acinetobacter baumannii* in one, organisms with intrinsic resistance to multiple antibiotic classes; pre-protocol isolates were predominantly *streptococci*, *enterococci*, and *Enterobacterales*, with *Pseudomonas aeruginosa* in one patient and *Candida parapsilosis* in another. Per-patient organisms, prophylaxis, and postoperative isolates are given in Table 3.

Postoperative cultures were obtained only when infection was clinically suspected, so postoperative microbiological data exist for the five infected patients alone. Among these, concordance between postoperative and preoperative isolates was complete in one, partial in two, absent in one, and not assessable in one for whom no preoperative culture was available. Because molecular strain typing was unavailable, species-level concordance cannot establish persistence of the same strain, and these observations should not be read as evidence of microbial persistence from the index wound.

### 3.5. Surgical Outcomes

**Figure 2:**
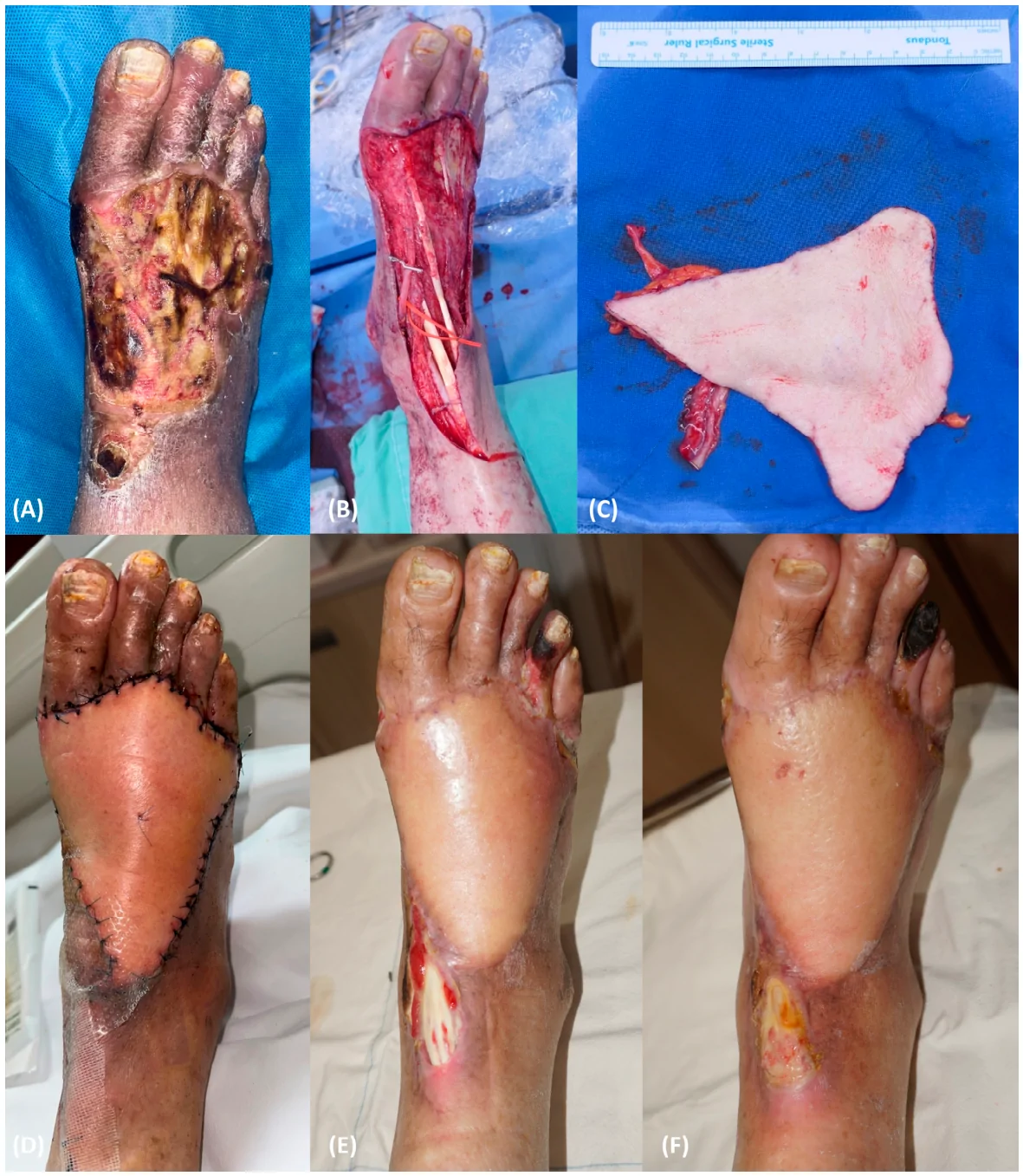
Representative case of microsurgical diabetic foot reconstruction. (**A**) Preoperative diabetic foot ulcer with exposure of vital structures; (**B**) surgical field after NaOCl antisepsis and debridement; (**C**) a free anterolateral thigh (ALT) flap was harvested; (**D**) one-week postoperative result; (**E**) three-months postoperative result; (**F**) five-months postoperative result.

The flap survival rate was 100% in both groups. Limb salvage was achieved in all 15 patients. The clinical course of each patient, including individual follow-up duration, infection type and onset, and new ulcer formation, is presented in Table 4. Flap revision surgery was required in one post-protocol patient (16.7%) and one pre-protocol patient (11.1%; *p* = 1.000). Both revisions were performed for arterial insufficiency due to arterial thrombosis, arising largely from underlying peripheral arterial disease; both patients had angiographically confirmed multilevel stenosis and had undergone revascularization before reconstruction. Both flaps were salvaged and both limbs preserved. Two post-protocol patients were identified as deceased during record review, at 4.4 months and at an unverified date. New ulcer formation during follow-up occurred in one post-protocol patient (16.7%) and in six of the eight pre-protocol patients with available follow-up (75.0%; OR 0.10, 95% CI 0.01–1.1; *p* = 0.103). A representative case—the preoperative wound, the surgical field after NaOCl antisepsis, and the postoperative reconstructive result—is shown in Figure 2.

## 4. Discussion

In this single-center feasibility study, the protocol was delivered as specified in all six post-protocol patients, within the standard operative workflow and without modification or abandonment of any planned procedure. Operative time did not differ materially between groups (median 388 versus 430 min; *p* = 0.596). Flap survival and limb salvage were achieved in every patient in both groups, including two flaps salvaged after revision; both revisions were undertaken for arterial insufficiency from thrombosis in limbs with established peripheral arterial disease. These outcomes compare with published series reporting flap survival around 92% and limb salvage of 83–85% [4,5,6], and indicate that the protocol did not adversely affect flap perfusion or healing and no adverse event attributable to the cleanser was observed. The protocol is therefore feasible in this setting, although the safety observation rests on six patients and is necessarily preliminary.

Turning to the descriptive secondary outcome, the total postoperative infection rate was 16.7% in the post-protocol group versus 44.4% in the pre-protocol group (OR 0.33, 95% CI 0.04–3.0; *p* = 0.580). No difference was demonstrated, and the confidence interval is compatible with benefit, no effect, or harm. The distribution of events is set out here as a description rather than an inference. Early infections (within 30 days) were comparable between groups (16.7% versus 11.1%, *p* = 1.000). Late infection (>30 days) was absent in the post-protocol group (0%) versus 33.3% in pre-protocol patients (*p* = 0.229); all three pre-protocol events were deep tissue infections at the reconstructed site, occurring at 5.0, 5.2, and 8.3 months. This distribution is consistent with persistence of organisms derived from the original wound, but the present data cannot establish that mechanism: no post-cleansing cultures were obtained, molecular strain typing was unavailable, and the comparison is confounded by markedly unequal observation time. In a landmark analysis restricted to a common six-month window and in incidence per person-time, no difference between groups was demonstrable.

The two groups were similar in all measured variables, including HbA1c, Wagner grade, BMI, five comorbidities, duration of diabetes (median 17 versus 8 years; *p* = 0.516), osteomyelitis (3/6 versus 5/9; *p* = 1.000), and revascularization (4/6 versus 4/9; *p* = 0.608), and carried comparable baseline culture positivity. Non-significant *p*-values in a sample of this size do not establish comparability, and residual confounding by unmeasured factors cannot be excluded. Ankle-brachial index was recorded but is reported with caution: arterial calcification, common in this population and confirmed on CT angiography, produces falsely elevated values. Perioperative antibiotic prophylaxis was heterogeneous and more frequently culture-directed in the post-protocol group, representing a potential era-related co-intervention. The residual age imbalance (median 70 versus 59 years) would be expected to bias against the post-protocol group.

The mechanistic rationale for targeting the wound bed in the perioperative window is based on biofilm pathophysiology. DFUs persisting beyond four weeks almost universally harbor bacteria in biofilm communities. Malone et al. documented biofilm in 78.2% of non-healing chronic wounds (95% CI 61.6–89.0%), with DFU prevalence likely higher given chronic hypoxia, impaired neutrophil function, repeated antibiotic exposure, and necrotic substrate [15]. The biofilm phenotype confers 100- to 1000-fold antimicrobial resistance compared with planktonic organisms through reduced metabolic activity, restricted antibiotic diffusion through the extracellular polymeric substance (EPS) matrix, efflux pump upregulation, and phenotypically distinct persister cells [16,17]. Surgical debridement reduces biofilm burden substantially but leaves residual organisms at wound margins and tissue crevices [6]. Once the wound is closed, the warm, nutrient-rich, relatively hypoxic flap environment permits residual biofilm to re-establish and emerge as late infection.

The choice of agent followed from these considerations. Povidone-iodine, the standard for skin preparation, is cytotoxic at bactericidal concentrations and is inactivated by the organic debris of an open diabetic foot wound. Sodium hypochlorite retains broad-spectrum oxidative activity in that environment, and its potency is pH-dependent: below the hypochlorous acid pKa of 7.5, the uncharged, membrane-permeant acid predominates, whereas above it, the poorly active hypochlorite ion dominates [18,19,20]. The preparation used here is formulated at near-neutral pH with betaine as a surfactant co-ingredient, a combination reported to retain antimicrobial activity at concentrations with comparatively low cytotoxicity to fibroblasts and keratinocytes [21]. It retains activity in the presence of organic debris better than povidone-iodine, though it is progressively consumed by organic load.

Of particular mechanistic relevance is the 2023 ex vivo investigation by Grealy et al., in which debrided DFU tissues from 30 patients with type 2 diabetic were immersed in pH-neutral HOCl (200 ppm) for three minutes, directly modeling the wound bed component of preoperative surgical field antisepsis [22]. Microbial loads were reduced by 1065-fold (1 mL) and 8216-fold (10 mL) versus saline controls (both *p* < 0.0005). Critically, 82% of predominantly non-infected DFU tissues harbored bacterial burdens above 10^5^ CFU/g, the threshold above which healing is clinically impaired, yet only 9.5% were clinically diagnosed as infected, substantiating the rationale for preoperative wound bed antisepsis even in the absence of overt clinical infection.

The principle that reducing microbial burden at the operative site immediately before incision lowers postoperative infection is well established in clean and clean-contaminated surgery. A randomized clinical trial shows that preoperative chlorhexidine-alcohol is more effective than povidone-iodine after clean-contaminated procedures (9.5% versus 16.1%) [23]; a network meta-analysis of 27 RCTs confirmed 2.0–2.5% chlorhexidine in alcohol as the most effective skin agent [10]; and a subsequent network meta-analysis of 41 RCTs (17,188 patients) found that antiseptic incisional irrigation reduces SSI (relative risk 0.60), whereas saline does not [11]. However, this evidence base applies to intact perilesional skin. The DFU presenting for reconstruction is a dirty or infected wound, the highest contamination category [24]; the International Wound Infection Institute (IWII) 2025 consensus recommends antimicrobial cleansing when infection is confirmed or suspected [25], criteria met by definition at the biofilm-established DFU reconstructive interface, yet published DFU reconstruction protocols specify no antiseptic for the open wound surface between final debridement and closure [14].

The skin of the diabetic foot and lower leg also differs fundamentally from skin elsewhere: it harbors higher commensal loads in ridged surfaces, nail folds, and web spaces, frequently covered by hyperkeratotic debris that physically obstructs antiseptic contact. Dingemans et al. showed that 0.5% chlorhexidine-in-alcohol alone is insufficient for foot and ankle antisepsis, with nailfold bacterial loads of a median of 92 CFU versus 1 CFU when an alcohol footbath was added (*p* = 0.03) [26]. The protocol described here addresses both elements: NaOCl with betaine disrupts wound-bed biofilm, while wipe/swab application across the foot and lower leg mechanically removes crust and hyperkeratotic debris that would otherwise mask perilesional skin from subsequent povidone-iodine draping. It differs from the nearest published analog—HOCl wound-bed preparation before flap closure of chronic pressure injuries [13]—both in the target wound (diabetic foot versus pressure injury) and in extending antisepsis to the entire surgical field before draping rather than irrigating the wound at closure.

Delayed infection after reconstruction of the diabetic foot is recognized clinically but poorly characterized, and this is the principal obstacle to interpreting the present data. Conventional surgical site infection surveillance applies a 30-day window, extended to 90 days where an implant is placed [24]; infection arising months after flap coverage therefore falls outside the interval in which surgical infection is conventionally ascertained, and reconstructive series report such events, when they report them at all, as osteomyelitis or ulcer recurrence over an undefined interval rather than as a timed surgical endpoint. In a pooled cohort of 171 patients treated for diabetic foot osteomyelitis, re-infection occurred in 27.5% within twelve months at a mean of 76 days from treatment, with published rates across studies ranging from 16.7% to 56.7% per year [27]. In the reconstructive literature, a series of 65 patients with lower extremity osteomyelitis treated with free flap, of whom 43% had diabetic foot ulcer as the underlying cause, reported osteomyelitis recurrence in 12.3% over a mean follow-up of 18 months, and the authors noted explicitly that their follow-up was too short because recurrence may occur years after treatment [28]. Against these figures, the three late episodes in our pre-protocol group are high but not aberrant, and the interval at which they arose, 5.0 to 8.3 months, lies well beyond conventional surveillance.

Whether such episodes represent persistence of the index infection or a new event cannot be resolved by the present data, and the distinction is not trivial. In a prospective surgical series of diabetic foot osteomyelitis occurring after healing, true recurrence occurred in 4.6% of patients, whereas ulcer recurrence occurred in 43% and new episodes of osteomyelitis in 16.9%, so new events outnumbered genuine recurrence by roughly four to one [29]. Diabetic foot ulcers themselves recur in a large proportion of patients within the first year [1]. New ulcer formation was observed in six of the eight pre-protocol patients with available follow-up in the present series, so ulcer recurrence with secondary infection is at least as plausible an explanation for the late episodes as persistence of organisms from the index wound. This reinforces rather than resolves the definitional problem raised above: without molecular strain typing, our late-infection category cannot separate these mechanisms, and we do not claim that it does.

Both cohorts were composed entirely of men. Although sex was not an eligibility criterion, every consecutive patient meeting the inclusion criteria during the study period was male. Thus, the composition reflects consecutive sampling rather than selection. This is consistent with the reported male predominance in diabetic foot ulceration, which becomes more marked at greater ulcer severity. In a Korean population-based cohort, 57.1% of patients with diabetic foot ulcer were male, and male sex was independently associated with amputation (hazard ratio 2.41) [30]. In a national cohort restricted to moderate-to-severe ulcers, 72% of patients were male, with deeper ulcers, more frequent probe-to-bone, and more frequent deep infection in men [31]. A meta-analysis of 20 studies found a pooled odds ratio for amputation of 1.68 (95% CI 1.31–2.15) in men [32]. A cohort drawn consecutively from the most severe end of the disease spectrum, as in here, would therefore be expected to be male-predominant. These findings nonetheless cannot be assumed to generalize to female patients, and sex-balanced enrollment should be specified prospectively in any future trial.

This study has several limitations inherent to a small, non-randomized feasibility design. The sample (n = 15) is small and the study underpowered; the between-group differences represent effect-size estimates rather than evidence of efficacy. The sequential before-and-after allocation confounds the intervention with calendar time, so a surgical learning curve or other unmeasured temporal changes in perioperative care cannot be excluded. Follow-up duration differed substantially between groups, limiting comparison of late infection over equivalent windows. Antibiotic prophylaxis was heterogeneous, and contact time was not prospectively standardized and could not be quantified from retrospective review. Molecular strain typing was unavailable, so persistence of organisms from the index wound cannot be demonstrated. Outcome ascertainment was unblinded, and one criterion for infection—the clinical decision to institute antibiotic therapy—was partly clinician-dependent, introducing potential detection bias. Vital status was not systematically ascertained: two patients in the protocol cohort were identified as deceased during record review, one confirmed at 4.4 months following cardiac arrest and one incidentally, with date and cause unverified. Deaths occurring outside our institution would not have been captured, and competing mortality contributes to the short observation period in the protocol cohort.

Notwithstanding these limitations, the study establishes that the protocol can be delivered within the standard operative workflow without adverse effect on reconstructive outcomes. A prospective randomized trial is required to determine efficacy. It should be powered on the late infection endpoint and should specify application parameters, including contact time and volume, paired pre- and post-cleansing quantitative wound cultures with molecular strain typing, blinded outcome adjudication, and uniform follow-up of at least 36 months with mortality treated as a competing risk.

## 5. Conclusions

This single-center feasibility study indicates that preoperative surgical-field antisepsis with a pH-optimized sodium hypochlorite (NaOCl)-based wound cleanser, applied to the wound bed and surrounding foot and lower leg before conventional povidone-iodine draping, is feasible in microsurgical diabetic foot reconstruction. No statistically significant difference in postoperative infection was demonstrated. The absence of late deep infection at the reconstructed site in the protocol cohort is hypothesis-generating and supports a powered randomized controlled trial incorporating paired pre- and post-cleansing quantitative wound cultures as a microbiological endpoint. With 100% flap survival and limb salvage in both groups, the protocol showed no adverse effect on reconstructive outcomes. To our knowledge, this is the first clinical evaluation of a whole-field preoperative antisepsis protocol addressing both the wound bed and the perilesional skin before draping in microsurgical diabetic foot reconstruction.

## Figures and Tables

**Table 1:** Patient demographics, comorbidities, and baseline microbiology (n = 15).

Variable	Post-Protocol (n = 6)	Pre-Protocol (n = 9)	*p*-Value
Demographics			
Age (yr), median (range)	70 (61–80)	59 (34–81)	0.181
BMI (kg/m^2^), median (range)	23.1 (17.6–27.6)	22.2 (18.3–41.7)	0.529
HbA1c (%), median (range)	8.3 (6.6–11.3)	8.2 (6.1–13.7)	0.860
DM duration (yr), median (range)	17 (4–42)	8 (3–26)	0.516
Comorbidities			
HTN, n (%)	3 (50%)	3 (33%)	0.622
Smoking, n (%)	3 (50%)	6 (67%)	0.622
PAD, n (%)	4 (67%)	4 (44%)	0.608
ESRD, n (%)	1 (17%)	2 (22%)	1.000
CAD, n (%)	2 (33%)	3 (33%)	1.000
Wound severity			
Wagner grade 2, n (%)	0 (0%)	1 (11.1%)	
Wagner grade 3, n (%)	3 (50%)	4 (44.4%)	
Wagner grade 4, n (%)	3 (50%)	4 (44.4%)	0.742
Osteomyelitis, n (%)	3 (50.0%)	5 (55.6%)	1.000
Perfusion			
ABI, median (range)	1.17 (0.49–1.27)	1.19 (0.55–1.33)	0.376
Angiographic lesion present, n (%)	4 (66.7%)	5 (55.6%)	1.000
Revascularization, n (%)	4 (66.7%)	4 (44.4%)	0.608
Microbiology			
Preoperative culture positive, n (%)	5 (83.3%)	7 (77.8%)	1.000

Abbreviations: HTN, hypertension; PAD, peripheral arterial disease; ESRD, end-stage renal disease; CAD, coronary artery disease. *p*-values by Fisher’s exact test (categorical) or Mann–Whitney *U* test (continuous and ordinal). Two pre-protocol patients had no preoperative culture on record. ABI, ankle-brachial index.

**Table 2:** Operative parameters, postoperative infection, and surgical outcomes.

Variable	Post-Protocol (n = 6)	Pre-Protocol (n = 9)	OR (CI)	*p*-Value
Operative parameters				
Flap size (cm^2^), median (range)	60 (28–120)	96 (32–240)	—	0.262
Op time (min), median (range)	388 (318–680)	430 (328–615)	—	0.596
Follow-up (m), median (range) *	4.5 (2–24)	24.5 (3–37)	—	0.060
Postoperative infection				
Early (≤30 days), n (%)	1 (16.7%)	1 (11.1%)	1.55 (0.13–18.9)	1.000
Late (>30 days), n (%)	0 (0.0%)	3 (33.3%)	0.14 (0.01–3.4)	0.229
Total infection, n (%)	1 (16.7%)	4 (44.4%)	0.33 (0.04–3.0)	0.580
Landmark analysis (≥6 m observed) ‡	0/3 (0.0%)	3/6 (50.0%)	0.14 (0.01–3.9)	0.464
Incidence per 100 person-months	2.27	2.33	—	—
Surgical outcomes				
Flap survival, n (%)	6 (100%)	9 (100%)	—	N/A
Limb salvage, n (%)	6 (100%)	9 (100%)	—	N/A
Flap revision, n (%)	1 (16.7%)	1 (11.1%)	—	1.000
New ulcer formation, n (%) §	1 (16.7%)	6 (75.0%)	0.10	0.103

Abbreviations: OR, odds ratio. *p*-values by Fisher’s exact test (categorical) or Mann–Whitney *U* test (continuous and ordinal). * Follow-up calculated for 8 of 9 pre-protocol patients; one patient lost to follow-up. ‡ Landmark analysis restricted to patients observed for at least 6 months, counting events occurring within that window. § New ulcer formation assessed in 8 of 9 pre-protocol patients; one patient lost to follow-up.

**Table 3:** Microbiological findings and perioperative antibiotic prophylaxis (n = 15).

Group	No.	Preoperative Culture	Perioperative Prophylaxis	Postoperative Culture	Species Concordance
Post-protocol	1	*Corynebacterium striatum*	Teicoplanin	Not obtained	NA
	2	*S. maltophilia*; *K. aerogenes*	Cefepime	*S. maltophilia*; *E. faecalis*	Partial
	3	Gram-positive rods	Ampicillin/sulbactam	Not obtained	NA
	4	*S. maltophilia*	TMP–SMX	Not obtained	NA
	5	No growth	Cefazolin + clindamycin	Not obtained	NA
	6	*A. baumannii*; other staphylococci	Ampicillin/sulbactam	Not obtained	NA
Pre-protocol	7	*P. aeruginosa*	Ampicillin/sulbactam + teicoplanin	Not obtained	NA
	8	*S. agalactiae*; *E. cloacae*	Ceftriaxone	*S. maltophilia*; *E. cloacae*; *A. lwoffii*	Partial
	9	MSSA	Ampicillin/sulbactam	Not obtained	NA
	10	*S. anginosus*	Ceftriaxone + levofloxacin	Not obtained	NA
	11	*Candida parapsilosis*; Gram-positive rods	Ampicillin/sulbactam	*S. marcescens*; *P. mirabilis*	None
	12	Not available	Flomoxef	*P. aeruginosa*	Not assessable
	13	Not available	Ampicillin/sulbactam	Not obtained	NA
	14	*E. raffinosus*; *E. faecalis*	Ampicillin/sulbactam	Not obtained	NA
	15	*S. agalactiae*	Ampicillin/sulbactam	*S. agalactiae*; *P. vulgaris*	Complete

Postoperative cultures were obtained only when infection was clinically suspected; NA, not applicable. Concordance compares postoperative with preoperative isolates: complete, all preoperative organisms recovered; partial, at least one organism shared; none, no organism shared; not assessable, no preoperative culture available. MSSA, methicillin-susceptible *Staphylococcus aureus*; TMP–SMX, trimethoprim–sulfamethoxazole, *A. baumannii*, *Acinetobacter baumannii*; *A. lwoffii*, *Acinetobacter lwoffii*; *E. cloacae*, *Enterobacter cloacae*; *E. faecalis*, *Enterococcus faecalis*; *E. raffinosus*, *Enterococcus raffinosus*; *K. aerogenes*, *Klebsiella aerogenes*; *P. aeruginosa*, *Pseudomonas aeruginosa*; *P. mirabilis*, *Proteus mirabilis*; *P. vulgaris*, *Proteus vulgaris*; *S. agalactiae*, *Streptococcus agalactiae*; *S. anginosus*, *Streptococcus anginosus*; *S. maltophilia*, *Stenotrophomonas maltophilia*; *S. marcescens*, *Serratia marcescens*.

**Table 4:** Per-patient clinical course and outcomes (n = 15).

Group	No.	Age	Date	Site	Flap	Size (cm^2^)	Op Time (min)	Flap Revision	New Ulcer	Follow-Up (m)	Infection (m)
Post-protocol	1	64	May. 2024	Foot	ALT	60	465	No	No	6	None
	2	79	Mar. 2024	Foot	VRAM	120	318	Yes	No	3	Early, 0.3 m
	3	76	Jan. 2024	Foot	MSAP	50	325	No	No	24	None
	4	80	Dec. 2023	Foot	ALT	120	680	No	Yes	6	None
	5	63	Nov. 2023	Foot	MSAP	60	445	No	No	3	None
	6	61	Nov. 2023	Foot	MSAP	28	330	No	No	2	None
Pre-protocol	7	66	Sep. 2023	Ankle	MSAP	75	550	No	Yes	17	None
	8	67	Jul. 2023	Foot	SIEA	240	595	Yes	Yes	33	Late, 5.0 m
	9	34	Jun. 2023	Heel	SIEA	98	430	No	Yes	10	None
	10	69	May. 2023	Foot	ALT	105	420	No	No	5	None
	11	46	Apr. 2023	Foot	SIEA	160	436	No	Yes	37	Early, 0.4 m
	12	59	Apr. 2023	Ankle	AMT	96	420	No	Yes	35	Late, 8.3 m
	13	43	Mar. 2023	Heel	MSAP	32	328	No	No	3	None
	14	81	Feb. 2023	Foot	ALT	78	615	No	NA	Lost	None
	15	50	Jan. 2023	Heel	MSAP	75	390	No	Yes	32	Late, 5.2 m

All patients were male. Flap survival and limb salvage were achieved in all 15 patients. Early infection occurred within 30 days and late infection beyond 30 days of reconstruction. Patient No. 14 was lost to follow-up at 3 months and contributed no further observation; NA, not assessable. ALT, anterolateral thigh; AMT, anteromedial thigh; MSAP, medial sural artery perforator; SIEA, superficial inferior epigastric artery; VRAM, vertical rectus abdominis myocutaneous.

## Data Availability

The data presented in this study are available on request from the corresponding author. The data are not publicly available because they consist of de-identified clinical records, the sharing of which is restricted by patient privacy considerations and the terms of IRB approval (HC26RISI0076).

## Supplementary Materials

The following supporting information can be downloaded at: https://www.mdpi.com/article/10.3390/jcm15166272/s1, Video S1: jcm-15-06272-supplemementary.mp4.

## Abbreviations

The following abbreviations are used in this manuscript:

ALT	Anterolateral thigh
AMT	Anteromedial thigh
BMI	Body mass index
CFU	Colony-forming units
DFU	Diabetic foot ulcer
EPS	Extracellular polymeric substance
HbA1c	Glycated hemoglobin
HOCl	Hypochlorous acid
IWII	International Wound Infection Institute
MSAP	Medial sural artery perforator
NaOCl	Sodium hypochlorite
OR	Odds ratio
RCT	Randomized controlled trial
SIEA	Superficial inferior epigastric artery
SSI	Surgical site infection
VRAM	Vertical rectus abdominis myocutaneous
